# C-Jun N-Terminal Kinase 2 Promotes Liver Injury via the Mitochondrial Permeability Transition after Hemorrhage and Resuscitation

**DOI:** 10.1155/2012/641982

**Published:** 2012-06-27

**Authors:** Christoph Czerny, Tom P. Theruvath, Eduardo N. Maldonado, Mark Lehnert, Ingo Marzi, Zhi Zhong, John J. Lemasters

**Affiliations:** ^1^Center for Cell Death, Injury & Regeneration, Departments of Pharmaceutical & Biomedical Sciences, Medical University of South Carolina, Charleston, SC 29425, USA; ^2^Departement of Trauma Surgery, J.W. Goethe University Frankfurt am Main, 60590 Frankfurt am Main, Germany; ^3^Biochemistry & Molecular Biology, Medical University of South Carolina, MSC 140, Charleston, SC 29425, USA

## Abstract

Hemorrhagic shock leads to hepatic hypoperfusion and activation of mitogen-activated stress kinases (MAPK) like c-Jun N-terminal kinase (JNK) 1 and 2. Our aim was to determine whether mitochondrial dysfunction leading to hepatic necrosis and apoptosis after hemorrhage/resuscitation (H/R) was dependent on JNK2. Under pentobarbital anesthesia, wildtype (WT) and JNK2 deficient (KO) mice were hemorrhaged to 30 mm Hg for 3 h and then resuscitated with shed blood plus half the volume of lactated Ringer's solution. Serum alanine aminotransferase (ALT), necrosis, apoptosis and oxidative stress were assessed 6 h after resuscitation. Mitochondrial polarization was assessed by intravital microscopy. After H/R, ALT in WT-mice increased from 130 U/L to 4800 U/L. In KO-mice, ALT after H/R was blunted to 1800 U/l (*P* < 0.05). Necrosis, caspase-3 activity and ROS were all substantially decreased in KO compared to WT mice after H/R. After sham operation, intravital microscopy revealed punctate mitochondrial staining by rhodamine 123 (Rh123), indicating normal mitochondrial polarization. At 4 h after H/R, Rh123 staining became dim and diffuse in 58% of hepatocytes, indicating depolarization and onset of the mitochondrial permeability transition (MPT). By contrast, KO mice displayed less depolarization after H/R (23%, *P* < 0.05). In conclusion, JNK2 contributes to MPT-mediated liver injury after H/R.

## 1. Introduction

Multiple trauma is the principal cause of hemorrhagic shock and is typically the consequence of traffic accidents, falls, and, in time of war, casualties of combat [1, 2]. After hemorrhagic shock, resuscitation can lead to multiple organ dysfunction syndrome (MODS), which remains the most significant contributor to late mortality and intensive care unit resource utilization in critical care medicine [3, 4]. The liver is quite vulnerable to injury after ischemia and reperfusion (I/R). After I/R, hepatic necrosis is the predominant mode of cell death, whereas apoptosis is of less importance [5–7]. However, apoptosis and necrosis share common pathways, particularly the mitochondrial permeability transition (MPT) [8]. 

The MPT is caused by opening of high conductance MPT pores in the mitochondrial inner membrane, which leads to mitochondrial depolarization, uncoupling of oxidative phosphorylation, and large amplitude mitochondrial swelling [9]. The MPT plays a prominent role in the pathogenesis of cell death after I/R injury and a variety of other stresses [9–12]. After onset of the MPT, necrotic cell killing (oncosis) can occur as a consequence of ATP depletion, whereas swelling of mitochondria after the MPT leads to rupture of the outer membrane and release of proapoptotic proteins like cytochrome c. The extent of ATP depletion is crucial to whether necrosis or apoptosis occurs, since caspase-dependent apoptosis requires ATP, and necrosis does not occur until ATP is depleted by more than 85%.

c-Jun N-terminal kinase (JNK) is a stress-activated protein kinase that becomes activated after stresses like ultraviolet (UV) radiation, I/R and inflammation [13–16]. JNK-dependent phosphorylation of the transcription factor c-Jun/AP-1 promotes gene expression for an enhanced immune response [17]. JNK can also induce apoptosis via JNK-mediated phosphorylation of proapoptotic Bcl2 family proteins, such as Bim and Bmf, leading to mitochondrial outer membrane permeabilization, release of cytochrome c, and caspase activation [18, 19]. Moreover, translocation of activated JNK to mitochondria promotes the MPT [20, 21]. JNK becomes activated after experimental liver transplantation, warm hepatic I/R and hemorrhage/resuscitation (H/R), and pharmacological inhibition of JNK decreases liver injury, improves liver function, and increases survival in these settings [14, 15, 22–25]. Liver expresses two isoforms of JNK—JNK1 and JNK2 [26]. In models of acetaminophen hepatotoxicity, TNF*α*-dependent hepatic injury, warm I/R to liver and liver transplantation, JNK2 deficient mice are relatively protected against injury compared to wildtype mice [27–30].

Another organ vulnerable to injury during H/R is the gut. H/R compromises the barrier function of the gut, causing toxins and bacterial products like lipopolysaccharide (LPS) to enter the liver via the portal vein [31]. LPS and other gut-derived toxins entering the liver after H/R stimulate free radical generation and proinflammatory cytokine release by Kupffer cells to contribute to hepatic injury and increased cytokines in the blood stream [32–35]. Since JNK2 is also associated with the loss of barrier function of the gut [36, 37], we hypothesized that JNK2 is important for promotion of liver injury after H/R. Here, we test this hypothesis and show that liver injury decreases and hepatic function improves after H/R to JNK2 deficient mice in comparison to wildtype mice. These improvements are associated with improved mitochondrial function.

## 2. Materials and Methods

### 2.1. Chemicals and Reagents

Rhodamine 123 (Rh123) and other reagents were purchased from Sigma-Aldrich (St. Louis, MO, USA).

### 2.2. Animals

Experiments were performed using protocols approved by the Institutional Animal Care and Use Committee. C57BL/6 (wildtype) and JNK2-deficient (B6.129S2-Mapk9tm1Flv/J on a C57BL background) mice were obtained from Jackson Laboratory (Bar Harbor, ME). All mice used were males of 8 to 10 weeks of age and weighing 21–25 g.

### 2.3. Hemorrhagic Shock and Resuscitation

After an overnight fast, mice were anesthetized with sodium pentobarbital (80 mg/kg body weight). Under spontaneous breathing, the left and right femoral arteries were exposed and cannulated with polyethylene-10 catheters (SIMS Portex), as described [15]. Before insertion, the catheters were flushed with normal saline containing heparin (100 IU/l). One catheter was connected via a transducer to a pressure analyzer (Micro-Med; Louisville, KY, USA), and blood was withdrawn over 5 min via the second catheter into a heparinized syringe (10 units) to a mean arterial pressure of 30 mm Hg. This pressure was maintained for 3 h by the reinfusion or withdrawal of shed blood. An animal temperature controller was used to maintain rectal temperature between 36.6 and 37.3°C. After 3 h, mice were resuscitated with the shed blood followed by lactated Ringer's solution corresponding to 50% of the shed blood volume infused with a syringe pump over 30 min. Adequacy of resuscitation was determined by the restoration of blood pressure to ~80 mm Hg. After resuscitation, the catheters were removed, the vessels were ligated, and the groin incisions were closed. Sham-operated animals underwent the same surgical procedures without hemorrhage. In sham-operated mice, pentobarbital anesthesia lasted up to 120 min before the animals began to awaken, and a second injection was required to continue the anesthesia. In mice undergoing H/R, a second injection of pentobarbital was not necessary to maintain anesthesia, most likely due to decreased pentobarbital metabolism by the hypoperfused liver. Over the course of the experiments, no mortality in any group occurred. For the determination of H/R-dependent liver damage, mice were anesthetized, and the two right dorsal liver lobes were snap frozen in liquid nitrogen. The remaining liver was flushed with saline through the portal vein, fixed by infusion of 4% buffered paraformaldehyde, and embedded in paraffin.

### 2.4. Alanine Aminotransferase (ALT)

 Blood samples to measure ALT were collected from the inferior vena cava 6 h after H/R for analysis using a kit (Sigma Chemical, St. Louis, MO, USA).

### 2.5. Histology

 Necrosis was evaluated 6 h after H/R in 4-*μ*m paraffin sections stained with hematoxylin and eosin (H&E). Necrosis was identified by standard morphologic criteria (e.g., loss of architecture, karyolysis, vacuolization, increased eosinophilia). Areas of necrosis were outlined in 10 random fields for each liver. Images were captured (Olympus BH-2 Microscope; Micropublisher 5.0 RTV, Center Valley, PA, USA), and the area percentage of necrosis was quantified using a computer program (BioQuant BQ Nova Prime 6.7, R&M Biometrics, Nashville, TN, USA).

### 2.6. Caspase-3

 Liver tissue (~100 mg) was homogenized (Polytron PT-MR2100, Kinematica, Luzern, Switzerland) in 1 mL of lysis buffer containing 0.1% 3[(3-cholamidopropyl)dimethylammonio]-propanesulfonic acid, 5 mM DTT, 2 mM EDTA, 1 mM pefabloc, 10 ng/mL pepstatin A, 10 ng/mL aprotinin, 20 *μ*g/mL leupeptin and 10 mM HEPES buffer, pH 7.4. After centrifugation at 15,000 rpm for 30 min, activity of caspase-3 in the supernatant was determined using a Caspase-3 Colorimetric Assay Kit (R&D Systems, Minneapolis, MN) according to the manufacturer's instructions. Activity was normalized to protein concentration and expressed as fold increase compared to sham.

### 2.7. 4-Hydroxynonenal

 Paraffin sections were deparaffinized, rehydrated, and incubated with polyclonal antibodies against 4-hydroxynonenal (4-HNE, Alpha Diagnostics; San Antonio, TX, USA) in PBS (pH 7.4) containing 1% Tween 20 and 1% bovine serum albumin. Peroxidase-linked secondary antibody and diaminobenzidine (Peroxidase Envision Kit, DAKO) were used to detect specific binding.

### 2.8. Intravital Microscopy

 At 4 h after H/R, mice were anesthetized with pentobarbital (50 mg/kg, i.p.) and connected to a small animal ventilator via a tracheostomy and respiratory tube (22-gauge catheter), as described [29]. Laparotomy was performed, and a polyethylene-10 catheter was inserted into the distal right colic vein. Using a syringe pump, a membrane potential indicating fluorophore, Rh123 (1 *μ*mol/mouse), was infused via the catheter over 10 min. After prone positioning of the mouse, the liver was gently withdrawn from the abdominal cavity and placed over a glass coverslip on the stage of an inverted microscope. Rh123 fluorescence was excited with 820 nm light from a Chameleon Ultra Ti-Sapphire pulsed laser (Coherent, Santa Clara, CA, USA) and imaged with a Zeiss LSM 510 NLO laser scanning confocal microscope using a 63 × 1.3 NA water-immersion objective lens. Green Rh123 fluorescence was collected through a 525 ± 25 nm band pass filter. During image acquisition, the respirator was turned off for ~5 sec to eliminate breathing movement artifacts. In 20 fields per liver, hepatocytes were scored for bright punctate Rh123 fluorescence signifying polarized mitochondria or a dimmer diffuse cytosolic fluorescence denoting depolarized mitochondria. Image analysis was performed in a blinded manner.

### 2.9. Statistical Analysis

 Data are presented as means ± S.E., unless noted otherwise. Statistical analysis was performed by ANOVA with Student-Newman-Keuls test, as appropriate, using *P* < 0.05 as the criterion of significance.

## 3. Results

### 3.1. Decreased ALT Release and Liver Necrosis after Hemorrhage and Resuscitation of JNK2-Deficient Mice

**Figure 1:**
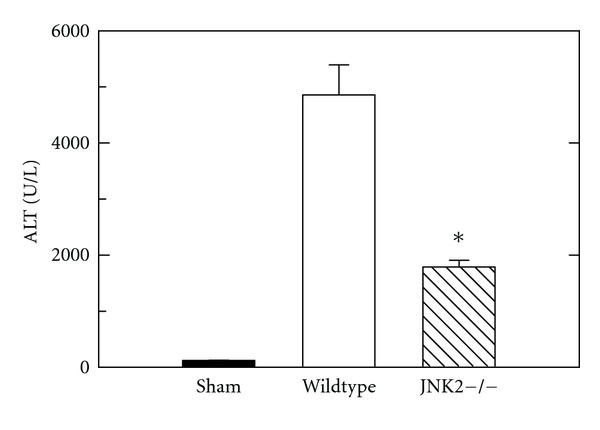
Decreased alanine aminotransferase (ALT) release after hemorrhage/resuscitation in JNK2-deficient mice. Wildtype and JNK2-deficient (JNK2−/−) mice were subjected to sham operation or bled to a mean arterial pressure of 30 mm Hg and resuscitated after 3 h, as described in Section 2. Blood was collected at 6 h after resuscitation for ALT measurement. Group sizes were 5-6 mice/group. *P < 0.05 versus wildtype. Average ALT values of wildtype and JNK2 deficient mice after sham operation were not statistically significantly different and are pooled.

 After sham operation, serum ALT averaged  112 ± 15 U/L in wildtype and JNK2 deficient mice (Figure 1). After H/R, ALT increased to  4860 ± 538 U/L 6 h after resuscitation in wildtype mice compared to  1806 ± 126 U/L in JNK2-deficient mice (*P* < 0.001, Figure 1).

**Figure 2:**
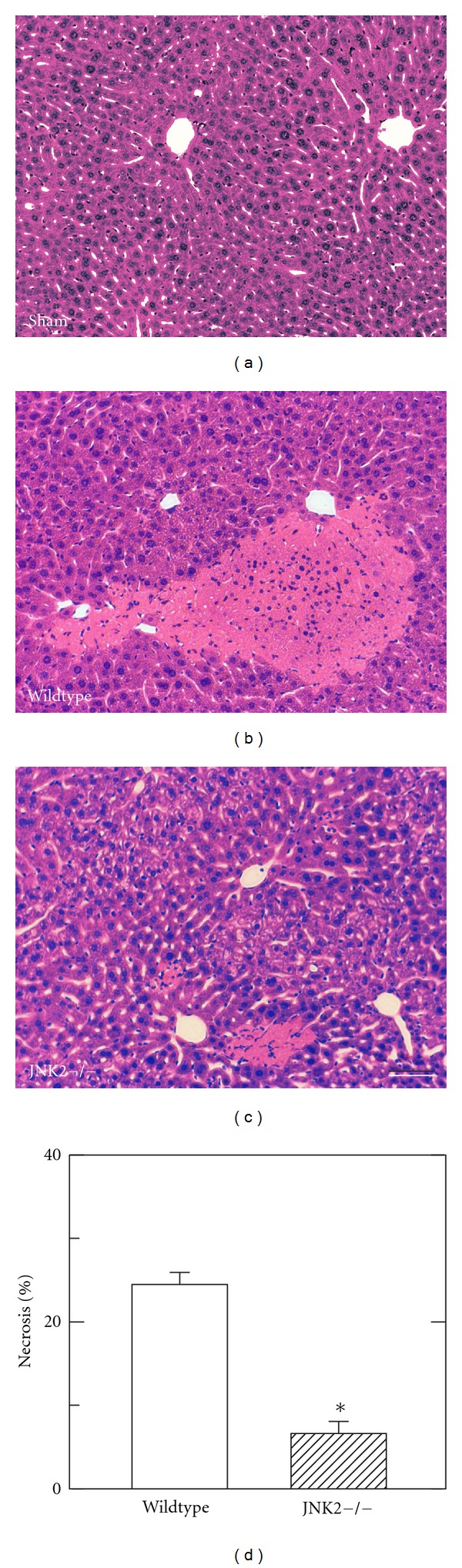
Decreased necrosis after hemorrhage and resuscitation in JNK2 deficient mice. At 6 h after resuscitation, necrosis was assessed by H&E in livers from sham-operated wildtype mice (a) and from wildtype- (b) and JNK2-deficient (c) mice after H/R. Bar is 50 *μ*m. In (d), the percent area of necrosis is averaged from 5 livers per group. Necrosis was not present after sham operation of either wildtype- or JNK2 deficient mice and is not plotted. *P < 0.05.

In sham-operated wildtype and JNK2-deficient mice, liver histology was normal and indistinguishable from untreated mice (Figure 2(a) and data not shown). At 6 h after H/R to wildtype mice, large areas of hepatic necrosis developed with a predominantly pericentral and midzonal distribution (Figure 2(b)). In JNK2-deficient mice, hepatic necrosis after H/R decreased from 24.5 ± 1.5% in wildtype mice to 6.6 ± 1.5% (*P* < 0.05, Figures 2(c) and 2(d)). Thus, hepatic necrosis in JNK2-deficient mice after H/R was decreased by more than two-thirds in comparison to wildtype mice (Figure 2(d)).

### 3.2. Decreased Apoptosis after Hemorrhage and Resuscitation of JNK2-Deficient Mice

**Figure 3:**
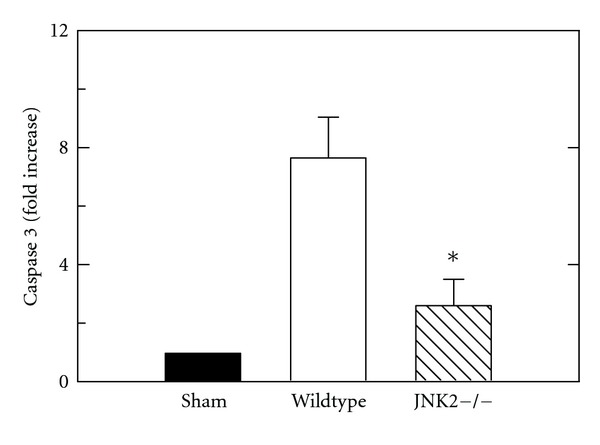
Decreased caspase 3 activation after hemorrhage and resuscitation of JNK2-deficient mice. At 6 h postoperatively, caspase 3 activity was assessed after sham operation and after H/R of wildtype and JNK2-deficient (JNK2−/−)mice, as described in Section 2. *P* < 0.05 versus wildtype, *n* = 5 per group.

 Caspase 3 activity was measured in liver extracts at 6 h after H/R of wildtype- and JNK2-deficient mice in comparison to sham-operated mice. After sham operation, caspase 3 activity in the liver was nearly undetectable (Figure 3). After H/R of wildtype mice, caspase 3 activity increased significantly by 7.6-fold. By contrast after H/R of JNK2-deficient mice, hepatic caspase 3 activity increased only 2.6-fold (*P* < 0.05 versus wildtype, Figure 3).

### 3.3. Improved Mitochondrial Function *In Vivo* after Hemorrhage and Resuscitation of JNK2-Deficient Livers

**Figure 4:**
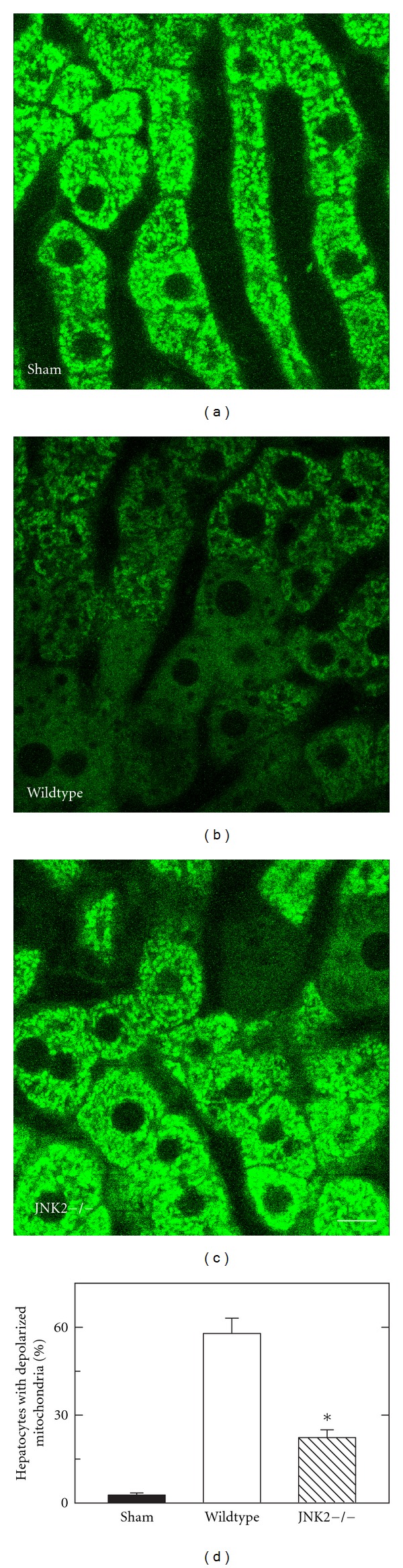
Decreased mitochondrial depolarization after hemorrhage and resuscitation of JNK2-deficient mice. Multiphoton imaging of hepatic Rh123 fluorescence was performed at 4 h after sham operation to wildtype mice (a) and H/R of wildtype- (b) and JNK2-deficient (c) mice, as described in Section 2. The percentage of hepatocytes per HPF with depolarized mitochondria is plotted in (d). Bar is 10 *μ*m. *P* < 0.05 versus other groups; *n* = 3 per group.

Intravital multiphoton microscopy revealed bright fluorescence of Rh123 in hepatocytes at 4 h after sham operation. The punctate pattern denoted polarization of individual mitochondria. No differences in Rh123 fluorescence were observed between livers of wildtype- and JNK2-deficient mice (Figure 4(a) and data not shown). We then imaged Rh123 fluorescence at 4 h after H/R. This time point was selected because previous studies of liver transplantation after cold ischemic storage showed that 4 h after reperfusion was a time point where mitochondrial dysfunction could be detected prior to onset of cell death [38]. At 4 h after H/R in wildtype mice, Rh123 staining became diffuse and dim in many hepatocytes indicative of depolarized mitochondria (Figure 4(b)). By contrast, after H/R of JNK2-deficient mice, mitochondria depolarized in fewer hepatocytes than in wildtype mice (Figure 4(c)). Rather, most hepatocytes exhibited bright, punctate staining by Rh123 in JNK2-deficient mice. In these experiments, hepatocytes were scored for Rh123 staining. In sham-operated mice, virtually no hepatocytes contained depolarized mitochondria. At 4 h after H/R of wildtype mice, 58% of hepatocytes contained depolarized mitochondria (Figure 4(d)). By contrast, at 4 h after H/R of JNK2-deficient mice, hepatocytes with depolarized mitochondria became 23%, less than half of that in wildtype mice (*P* < 0.05 versus wildtype, Figure 4(d)).

### 3.4. Decreased Oxidative Stress after Hemorrhage and Resuscitation of JNK2-Deficient Mice

**Figure 5:**
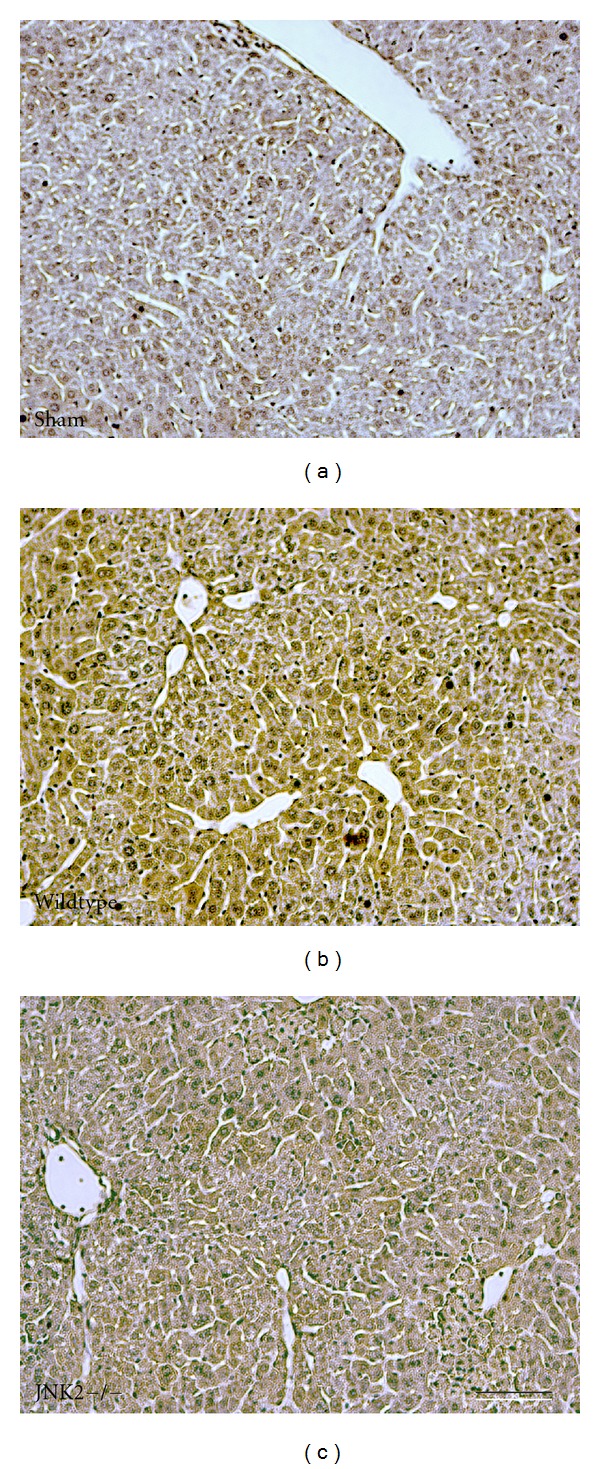
Decreased 4-hydroxynonenal immunostaining after hemorrhage and resuscitation of JNK2-deficient mice. ROS generation was assessed by 4-hydroxynonenal immunocytochemistry livers at 6 h after sham operation of wildtype mice (a) and after H/R to wildtype- (b) and JNK2-deficient (c) mice, as described in Section 2. Bar is 50 *μ*m. *n* = 5 per group.

 We used 4-HNE immunohistochemistry to evaluate oxidative stress in mouse livers 6 h after H/R. 4-HNE is a product of lipid peroxidation that forms protein adducts that are recognized by anti-4-HNE antibodies. After sham operation, the brown reaction product of 4-HNE immunohistochemistry was virtually undetectable (Figure 5(a)). By contrast at 6 h after H/R of wildtype mice, wide confluent areas of HNE immunoreactivity developed in pericentral and midzonal areas with relative sparing the periportal regions (Figure 5(b)). After H/R of JNK2-deficient mice, HNE immunoreactivity was substantially decreased and confined mostly to pericentral regions (Figure 5(c)). 

## 4. Discussion

### 4.1. Decreased Liver Injury after Hemorrhagic Shock and Resuscitation of JNK2-Deficient Mice

Systemic inflammatory response syndrome (SIRS) and MODS following H/R are major problems after multiple trauma [3, 4]. H/R also causes hepatic necrosis and apoptosis [15, 23, 39]. The goal of this study was to evaluate the impact of JNK2 on hepatic injury and mitochondrial dysfunction after H/R. Our findings show a specific role for JNK2 in liver injury after H/R, since JNK2-deficient mice had decreased hepatic injury and mitochondrial dysfunction after H/R in comparison to wildtype mice (Figures 1–4).

### 4.2. Reperfusion Injury after Hemorrhagic Shock and Resuscitation Induces Necrosis and Apoptosis through JNK2 Signaling

 JNK becomes activated in various models of liver injury, and pharmacological inhibition of JNK decreases liver injury [14, 15, 22–24, 40–42]. In particular, JNK inhibition with the peptide inhibitor, DJNKI-1, decreases hepatic damage and inflammation after H/R [23]. However, JNK inhibitors are nonspecific with regards to the two isoforms of JNK, JNK1 and JNK2, that are expressed in liver. Previous studies show that injury after orthotopic mouse liver transplantation and warm hepatic I/R decreases in JNK2-deficient livers compared to wildtype [29, 30]. In H/R, the specific roles of JNK isoforms are unknown. Therefore, we investigated the role of JNK2 by comparing JNK2-deficient mice and wildtype mice. 

JNK2 deficiency decreased both necrosis and apoptosis in liver after H/R. Necrosis assessed by ALT and histology and apoptosis assessed by caspase 3 activity were decreased by 60% or more in JNK2-deficient mice compared to wildtype (Figures 1 and 2). Nonetheless, necrosis was the predominant mode of cell death after H/R. These results are in agreement with earlier results after liver transplantation and warm I/R [29, 30].

### 4.3. JNK2 Deficiency Attenuates Formation of Reactive Oxygen Species after Hemorrhage and Resuscitation

Reactive oxygen species (ROS) mediate, at least in part, liver injury after H/R, warm I/R, and storage/reperfusion injury occurring in liver transplantation. A consequence of ROS formation is peroxidation of polyunsaturated fatty acids, such as linoleic and arachidonic acids, which leads to 4-HNE generation and formation of 4-HNE-protein adducts. In the present study, hepatic 4-HNE immunostaining was marked after H/R to wildtype mice but substantially diminished in JNK2-deficient mice (Figure 5). This indicates that JNK2 signaling has a role in promoting ROS generation after H/R. Such ROS can directly damage proteins, lipids, and DNA, as well as to help induce the MPT.

### 4.4. JNK2 Signaling after H/R Induces Mitochondrial Depolarization and Promotes Liver Injury

 To test the hypothesis that the JNK2 isoform specifically promotes mitochondrial dysfunction after H/R, we used intravital multiphoton microscopy of Rh123 to assess mitochondrial polarization. This technique allows direct assessment of mitochondrial polarization in livers of living animals. Four hours after H/R of wildtype livers, mitochondrial depolarization occurred in more than 50% of hepatocytes. Mitochondrial depolarization occurred prior to cell death, since after 4 h few cells labeled with propidium iodide, a marker of nonviable cells (data not shown), as described previously [29]. After H/R of JNK2-deficient mice, mitochondrial depolarization was markedly decreased in comparison to wildtype mice (Figure 4). Minocycline and N-methyl-4-isoleucine cyclosporin are specific inhibitors of the MPT that prevent mitochondrial depolarization after I/R and orthotopic rat liver transplantation with no direct effect on mitochondrial respiration and oxidative phosphorylation [29]. Thus, mitochondrial depolarization visualized by intravital multiphoton microscopy, which was attenuated in JNK2-deficient mice, most likely represents onset of the MPT. Several studies indicate involvement of the MPT in acetaminophen hepatotoxicity [12, 20]. In acetaminophen hepatotoxicity, activated JNK translocates to mitochondria to induce MPT onset, which can be prevented by JNK inhibitors [20]. Thus, protection against mitochondrial depolarization in JNK2-deficient livers after H/R implies that JNK2 is directly involved in promoting the MPT in wildtype livers after H/R stress.

### 4.5. Other Mechanisms Promoting JNK2-Dependent Toxicity

 H/R is also associated with a proinflammatory milieu in the gut lumen that promotes loss of barrier function [31]. Moreover, JNK2 mediates osmotic stress-induced tight junction disruption in the intestinal epithelium [36], although JNK1 is reported to mediate apical junction disassembly triggered by calcium depletion [37]. Impaired intestinal barrier function promoted by JNK during H/R may therefore also lead to portal vein endotoxemia, activation of TLR4 with phosphorylation of MAPKs, and increased production of inflammatory cytokines and ROS by hepatic Kupffer cells [34, 35, 43, 44]. Future studies will be needed to characterize how JNK2-dependent actions inside and outside hepatocytes contribute causally to liver injury, mitochondrial dysfunction, and development of MODS/SIRS after H/R.

### 4.6. Therapeutic Implications

 An important implication of the present findings is that JNK2 represents a unique therapeutic target for treatment and prevention of hepatic injury and possibly SIRS and MODS after H/R. D-JNKI-1 and other existing JNK inhibitors are nonspecific and inhibit all JNK isoforms: JNK1, JNK2, and JNK3 [45]. JNK2 in our model of H/R plays a detrimental role, but JNK1 and/or JNK3 may have beneficial effects in liver and other tissues, especially since JNK1/JNK2 double knockout mice are not viable [46]. Thus, a specific JNK2 inhibitor might provide greater and more specific benefit after H/R and decrease the potential of toxicity by JNK1 and/or JNK3 inhibition, but such an inhibitor still awaits development.

## List of Abbreviations

4-HNE: 4-hydroxynonenal
ALT: Alanine aminotransferase
H/R: Hemorrhage and resuscitation
H&E: Hematoxylin and eosin
HEPES: 4-(2-hydroxyethyl)-1-piperazineethanesulfonic acid
HPF: High-power field
MODS: Multiple organ dysfunction syndrome
MPT: Mitochondrial permeability transition
Rh123: Rhodamine 123
ROS: Reactive oxygen species
SIRS: Systemic inflammatory response syndrome.
